# Interventions delivered in healthcare settings to promote vaping cessation in children and young people (under the age of 18 years): a scoping review protocol

**DOI:** 10.1136/bmjopen-2025-102261

**Published:** 2025-09-08

**Authors:** Melvin Hoo Chuin Shen, Melissa Mae Gabriel, Louise Brennan, Rachel Isba

**Affiliations:** 1 Faculty of Health and Medicine, Lancaster University Medical School, Lancaster, UK; 2 Department of Paediatrics, Lancashire Teaching Hospitals NHS Foundation Trust, Preston, UK; 3 Population Health Sciences Institute, Newcastle University Faculty of Medical Sciences, Newcastle upon Tyne, UK; 4 Medical Services Directorate, Alder Hey Children’s Hospital, Liverpool, UK

**Keywords:** Child, Adolescent, Delivery of Health Care, Integrated, Smoking Reduction

## Abstract

**ABSTRACT:**

**Introduction:**

Vaping among children and young people (CYP) has increased globally over the past decade, with rates stabilising in the UK in recent years. Factors such as curiosity, social influence, stress management and attractive flavours contribute to its popularity. Although the long-term health impacts are uncertain, vaping poses risks including nicotine dependence, cardiovascular and respiratory issues, and cognitive impairment, though evidence on long-term effects is still emerging. Despite established smoking cessation programmes for adults, tailored resources for vaping cessation among CYP remain scarce, particularly within healthcare settings, which offer unique opportunities for professional intervention and ongoing support. The objective of this review is to assess the extent and nature of available literature on interventions delivered in healthcare settings to support vaping cessation among CYP under the age of 18 years.

**Methods and analysis:**

This scoping review will include studies targeting CYP under the age of 18 years, specifically focusing on interventions delivered within healthcare settings. Studies outside healthcare contexts or those without healthcare provider involvement will be excluded. Additionally, interventions delivered solely to parents or carers will not be considered.

A comprehensive search will be conducted in MEDLINE, Embase, Web of Science, Cochrane Library and CINAHL from January 2004 to present, with additional grey literature from sources including grey literature repositories and Google Scholar. Results will be imported into Rayyan for screening, with two independent reviewers assessing studies for inclusion. Data extraction will include study design, population characteristics (including explicit age ranges, specifically CYP under 18 years), intervention details and outcomes. A descriptive synthesis will map study characteristics, while thematic analysis will identify intervention themes and healthcare contexts.

**Ethics and dissemination:**

Ethics approval is not required for this secondary analysis. Findings will be disseminated through publication, conference presentations and shared with public health stakeholders.

**Strengths and limitations of this study**

- By focusing on the healthcare settings and interventions involving healthcare providers, this review highlights an underexplored area while acknowledging that it may not cover all contexts of vaping cessation support.
- The search strategy includes five electronic databases with peer-reviewed sources, as well as a range of grey literature sources.
- Stakeholders, including patient networks, will be engaged throughout the study.
- Limiting the review to English-language publications is a practical decision based on resource constraints, though it may result in the exclusion of some studies published in other languages.

## Introduction

 E-cigarette use, or vaping, has grown among children and young people (CYP) globally over the past decade with recent data indicating that rates in the UK have stabilised after earlier increases.^1 2^ In this paper, children and young people refer to individuals from birth until their 18th birthday, as defined by the General Medical Council (GMC) of the UK.^3^ While e-cigarettes are often marketed as smoking cessation aids for adults,^4^ they have also gained popularity among young, non-smoking populations due to a combination of factors, including curiosity, social influence and attractive flavours.^5 6^ Additionally, stress and mental health management are emerging reasons for vaping among CYP aged 11–17 years.^7^

Recent data collected by tobacco control charity Action on Smoking and Health (ASH) revealed that 18% of 11–17 years had tried vaping in 2024, compared with 20% in 2023, while current (defined as use in the past 30 days) vaping rates remain at 7.2%, unchanged since 2022.^7^ Globally, e-cigarette use among CYP is also significant; a meta-analysis in 2021 examining data from 69 countries found that 17.2% of CYP under the age of 20 years had tried vaping, while 7.8% were current users.^8^ Both the WHO and the US Surgeon General have flagged youth vaping as a significant public health concern,^9 10^ citing risks such as nicotine dependence, impairment of brain development, cardiovascular and respiratory issues, although evidence on long-term effects is still emerging.^11^,^13^

In the UK, the proposed 2024 Tobacco and Vapes Bill highlights concerns over youth vaping and suggests measures such as banning single-use vapes, implementing a retail licensing scheme, extending outdoor smoking bans and restricting packaging and flavours to make vaping products less appealing to young people.^14^ The proposed restrictions on packaging focus on reducing the attractiveness of designs and limiting branding to discourage uptake among children and young people. However, these measures are still under consideration and have yet to be passed into law.^14^

Despite the existence of established smoking cessation programmes for adults, evidence for effective strategies tailored specifically for CYP remains limited. Research underscores a lack of rigorously tested interventions aimed at CYP, leaving a significant gap in evidence-based cessation support for this age group.^15 16^ Behavioural approaches like motivational interviewing and cognitive-behavioural therapy have shown promise but require more robust evaluation for younger populations.^17 18^ Similarly, nicotine replacement therapy (NRT) and non-nicotine pharmacotherapies, such as bupropion and varenicline, need more investigation to establish their safety and efficacy for younger age groups.^19 20^ A Cochrane systematic review on interventions for vaping cessation in this age group found no eligible randomised controlled trials to date, emphasising the lack of evidence, though ongoing studies may address this gap in the future. In recognising the specific needs of CYP, organisations such as the WHO and the Office for Health Improvement and Disparities (formerly covered by Public Health England) now advocate for tailored cessation strategies that address both the psychological allure of e-cigarettes and the physical dependency on nicotine, which can develop rapidly in CYP.^9 21^

Healthcare settings present a unique opportunity for CYP vaping cessation interventions. Unlike community-based or school programmes, healthcare environments enable structured, ongoing screening and intervention by trained professionals, who can adapt existing tobacco cessation tools for e-cigarette use in CYP.^15^ Although the ongoing Cochrane review by Barnes *et al* included healthcare settings within its scope, none of the eligible completed studies were conducted in this environment. This may reflect the strict inclusion criteria of the review, which excluded non-randomised sources or grey literature, such as government reports, evaluations of community health programmes or data from smoking cessation initiatives that have not been formally published in academic literature.^16^ Of the 22 ongoing studies identified in the review, only four were conducted in healthcare settings, with the majority focusing on school-based or text messaging interventions.^16^ This suggests that healthcare environments remain underexplored for vaping cessation interventions among children and young people. The WHO identifies healthcare settings as a critical intervention point for cessation support, recommending the integration of e-cigarette cessation support into existing healthcare services.^9^ Routine interactions between healthcare providers and CYP create natural opportunities to screen for vaping behaviour, assess the severity of use and implement early interventions.^22^

This scoping review will contribute evidence towards addressing the gap in understanding effective, tailored approaches for helping CYP quit vaping. A scoping review was selected instead of a systematic review as it allows for a broader exploration of the literature, including all study designs and studies without formal designs. This approach is particularly suited to addressing gaps in the evidence base by capturing a wide range of interventions, including those reported in non-traditional sources.

A preliminary search of MEDLINE, the Cochrane Database of Systematic Reviews and Joanna Briggs Institute (JBI) systematic review register was conducted and no current or underway systematic reviews or scoping reviews on the topic were identified. This protocol adheres to the JBI methodology for conducting scoping reviews,^23 24^ by using the PRISMA-ScR (Preferred Reporting Items for Systematic reviews and Meta-Analyses extension for Scoping Reviews) checklist and guidelines.^25^

### Objective

The objective of this review is to identify and collate the available evidence, and to produce an overview of interventions delivered in healthcare settings with the aim of supporting CYP under the age of 18 to stop vaping.

## Methods and analysis

The proposed scoping review will be conducted in accordance with the JBI methodology for scoping reviews.^23^ The scoping review is planned to commence in August 2025, with anticipated completion by December 2025.

### Inclusion and exclusion criteria

Participants will include CYP who currently use e-cigarettes, defined as use within the past 30 days, as reported by ASH.^7^ For the purposes of this review, CYP are defined in accordance with the GMC as individuals from birth until their 18th birthday.^3^ The threshold of 18 years of age was chosen as this is the age under which individuals are legally considered children in the UK, according to the Children Act 1989.^26^ Studies involving mixed-age populations will be included if data specific to participants under 18 years can be extracted separately. Interventions delivered solely to parents or carers will be excluded to maintain a focus on interventions directly targeting the intended population.

This review will consider studies that explore interventions that specifically promote vaping cessation in healthcare settings. Interventions may include, but are not limited to, behavioural support (eg, motivational interviewing, cognitive behavioural therapy), pharmacological treatments such as NRT and non-nicotine medications (eg, bupropion, varenicline) and digital interventions (eg, text messaging support, telemedicine counselling).

Eligible interventions must be delivered by healthcare professionals, such as doctors, nurses or allied health practitioners, in clinical or community healthcare settings. Interventions in schools or community settings will be excluded unless they involve a healthcare provider (eg, school nurse) directly or are delivered in a healthcare-associated context. Studies conducted in healthcare settings from any country will be included to ensure a comprehensive understanding of global practices and interventions.

### Types of sources

This scoping review will consider qualitative and quantitative study designs, including both peer-reviewed sources and grey literature. Grey literature will encompass policy guidelines, government reports, organisational reports and other relevant non-peer-reviewed materials identified through grey literature repositories and targeted Google searches.

Eligible sources include experimental and quasiexperimental designs (eg, randomised controlled trials, non-randomised controlled trials, before-and-after studies and interrupted time-series studies), as well as analytical observational studies (eg, prospective and retrospective cohort studies, case-control studies, and analytical cross-sectional studies). Descriptive observational study designs, such as case series, individual case reports and descriptive cross-sectional studies, will also be included. Text and opinion papers are also considered for inclusion.

Sources in English from 2004 onward will be included, as e-cigarettes were first commercially available in that year.^27^ The restriction to English-language publications is due to practical considerations, including resource constraints related to translation and interpretation.

### Search strategy

The search strategy will aim to locate both published and unpublished studies, including peer-reviewed academic articles, reports, policy documents, theses, conference abstracts and other forms of grey literature. An initial limited search of MEDLINE was conducted to identify key articles on the topic. The text words in the titles and abstracts of relevant articles, along with the index terms used to describe them, were analysed to inform a full search strategy for Medline (see online supplemental appendix I). This search strategy, which includes all identified keywords and index terms, will be adapted for each database.

Sources published from January 2004 in English and indexed in MEDLINE, Embase, Web of Science, Cochrane Library and CINAHL will be searched. To capture grey literature and other relevant information, additional searches will be conducted using grey literature repositories and organisational websites relevant to public health, such as the NIH Repositories, WHO and CDC. Google will be used to locate local authority reports, third-sector publications and nonacademic documents that may not be indexed in traditional databases.

### Evidence selection

Following the search, all identified citations will be collated and uploaded into EndNote 20 (Clarivate Analytics, PA, USA), where duplicates will be removed. Titles and abstracts will be screened by two independent reviewers to assess eligibility based on the inclusion criteria for this review. Potentially relevant sources will be retrieved in full, with citation details imported into Rayyan, a web-based tool for managing and screening review articles, where duplicates will be removed.^28^

The full text of selected citations will then be evaluated against the inclusion criteria by two or more independent reviewers. Reasons for excluding studies at the full-text stage will be recorded and reported in the scoping review. Any disagreements arising between reviewers at any stage of the selection process will be resolved through discussion or consultation with an additional reviewer if necessary. The results of the search and selection process will be presented in full in the final scoping review, including a PRISMA flow diagram to detail the number of studies screened, included, and excluded at each stage.^25^

### Data extraction

Data will be extracted from sources included in the scoping review by two independent reviewers using a data extraction tool developed by the reviewers. A draft version of the extraction form based on JBI recommendations is provided (see online supplemental appendix II). The draft data extraction tool will be modified and revised if necessary, during the process of extracting data from each included source. Modifications to the tool during the extraction process will be documented and described in the final review.

The data extracted will include specific details about the participants, concept, context, study methods and key findings relevant to the review questions. Any disagreements that arise between reviewers during data extraction will be resolved through discussion or, if necessary, with the involvement of an additional reviewer. If required, authors of included studies will be contacted to provide missing or additional data.

### Data analysis and presentation

Descriptive data will be summarised and tabulated to provide an overview of source characteristics and intervention types. Qualitative synthesis will focus on identifying common themes regarding intervention efficacy, types of healthcare settings and contextual factors influencing intervention delivery. Data will be presented in a narrative format, accompanied by tables and charts where appropriate to provide a comprehensive overview of the current evidence on CYP vaping cessation interventions in healthcare settings. If sufficient data is available, subgroup analyses will be conducted to explore potential variations in findings according to participant characteristics, intervention types or healthcare settings.

### Patient and public involvement

While children and young people were not directly involved in this scoping review, the review is part of a larger project which arose from a Patient and Public Involvement and Engagement session with young people and which includes an ongoing programme of engagement.

### Ethics and dissemination

Ethics review was not required as this protocol involves secondary analysis of publicly available data. No patients or members of the public were directly involved in the design, conduct or reporting of this protocol. Findings will be submitted to a peer-reviewed journal. It could also be delivered in conference presentations and shared with public health stakeholders to inform future interventions and policies on CYP vaping cessation.

## Supplementary material

10.1136/bmjopen-2025-102261 online supplemental file 1
