# Supplementary material for: Interventions delivered in healthcare settings to promote vaping cessation in children and young people (under the age of 18 years): a scoping review protocol
Source: BMJ Open. 2025 Sep 8;15(9):e102261. doi: 10.1136/bmjopen-2025-102261 (PMC12421161; doi:10.1136/bmjopen-2025-102261)
Supplement: online supplemental file 1 [file bmjopen-15-9-s001.docx]

### **APPENDICES**

**Appendix I: Search strategy**

MEDLINE. Search date: February 2025

| **Search** | **Title/Abstract** | **MeSH Terms** | **Records Retrieved** |
| --- | --- | --- | --- |
| #1 | e-cig* OR "electronic cigarette*" OR vaping OR vape OR vapori* OR e-nicotine OR e-liquid | "Vaping"[MeSH] OR "Electronic Nicotine Delivery Systems"[MeSH] | 34,317 |
| #2 | cessation OR quit* OR stop* OR intervention OR management OR treatment OR “smoking cessation*” OR “nicotine replacement therap*” OR "smoking cessation agent*" OR "behavior therap*" OR “Health Education” OR “health promotion” | "Smoking Cessation"[MeSH] OR "Nicotine Replacement Therapy"[MeSH] OR "Smoking Cessation Agents"[MeSH] OR "Behavior Therapy"[MeSH] OR "Counseling"[MeSH] OR “Health Education”[MeSH] OR “Health Promotion”[MeSH] | 21,002,568 |
| #3 | adolescen* OR teen* OR youth* OR child* OR “young people” OR youth* OR “high school*” or “middle school*” OR “primary school*” OR “elementary school*” | "Adolescent"[MeSH] OR "Child"[MeSH] OR "Pediatrics"[MeSH] | 4,774,920 |
| #4 | clinic* OR hospital OR healthcare OR "health care" OR "primary care" OR "secondary care" OR "tertiary care" | "Primary Health Care"[MeSH] OR "Secondary Care"[MeSH] OR "Tertiary Healthcare"[MeSH] OR "Ambulatory Care Facilities"[MeSH] OR "Health Personnel"[MeSH] | 13,894,698 |
| #5 | #1 AND #2 AND #3 AND #4 |  | 2,984 |
| **Limit** | Limited to 2004 to present, in English | | |

**Appendix II: Data extraction instrument**

| **Scoping Review Details** | |
| --- | --- |
| Scoping Review Title | A scoping review of interventions delivered in healthcare settings to promote vaping cessation in children and young people (CYP). |
| Review Objective(s) | To identify and collate the available evidence, and to produce an overview of interventions delivered in healthcare settings with the aim of supporting CYP under the age of 18 to stop vaping. |
| Review Question(s) | What is the scope of existing evidence on vaping cessation interventions delivered in healthcare settings to support cessation among children and young people under 18 years of age? |
| **Inclusion/Exclusion Criteria** | |
| Population | Children and young people (aged less than 18 years) who currently use e-cigarettes (defined as use in the past 30 days). |
| Context | Interventions to promote vaping cessation delivered in healthcare settings. |
| Types of Study | Qualitative and quantitative studies, including experimental, quasi-experimental, observational, and descriptive study designs, as well as grey literature and policy reports. |
| **Study Details and Characteristics** | |
| Study Citation Details |  |
| Study Design |  |
| Country |  |
| Setting (e.g., primary care clinic, hospital, outpatient services) |  |
| Participants (e.g., age, gender, socioeconomic factors) |  |
| Population Sub-Group |  |
| **Details/Results Extracted from Study** | |
| Intervention |  |
| Outcome |  |
| Effectiveness |  |
