# Supplementary material for: Interventions delivered in healthcare settings to promote vaping cessation in children and young people (under the age of 18 years): a scoping review protocol
Source: BMJ Open. 2025 Sep 8;15(9):e102261. doi: 10.1136/bmjopen-2025-102261 (PMC12421161; doi:10.1136/bmjopen-2025-102261)
Supplement: Supplementary file 2 [file bmjopen-2025-102261.reviewer_comments.pdf]

PEER REVIEW HISTORY

BMJ Open publishes all reviews undertaken for accepted manuscripts. Reviewers are asked to complete a checklist review form and are provided with free text boxes to elaborate on their assessment. These free text comments are reproduced below.

ARTICLE DETAILS

Title (Provisional)

Interventions delivered in healthcare settings to promote vaping cessation in children and young people (under the age of 18 years): a scoping review protocol

Authors

Hoo Chuin Shen, Melvin; Gabriel, Melissa Mae; Brennan, Louise; Isba, Rachel

---

VERSION 1 - REVIEW

**Reviewer**	**1**
**Name**	**Sreedevi, Aswathy**
**Affiliation**	**Amrita Institute of Medical Sciences, Community Medicine**
**Date**	**13-Jul-2025**
**COI**	**None**

The authors present a very interesting area of work. This is the need of the hour with an increasing proportion of youngsters taking up vaping. It is very well written. I inly have a few minor comments.

1. Line 45-population characteristics ? should specify age group

2. Inclusion and exclusion criteria are well defined

4. Are health care environments underexplored in the Barnes study or there aren't any

6. All countries will be considered?.. please mention

7. Appendix1 -Pl check spelling of adolescent

8. Analysis: apart from descriptive ..subgroup analysis or sensitivity analysis could also be considered.

9. Is the scoping review registered?

VERSION 1 - AUTHOR RESPONSE

Reviewer Comment 1: Line 45 – Population characteristics? Should specify age group
Response: We clarified this by explicitly specifying the age group.

Action Taken: Revised text now states: "Data extraction will include study design, population characteristics (including explicit age ranges, specifically CYP under 18 years), intervention details, and outcomes."

Reviewer Comment 2: Inclusion and exclusion criteria are well defined
Response: Thank you. No changes were required.

Reviewer Comment 3: Are healthcare environments underexplored in the Barnes study, or are there no studies at all?
Response: The Cochrane review by Barnes et al. (2023) included healthcare settings within its scope. However, none of the eligible completed studies were conducted in these environments. This may reflect the strict inclusion criteria used in the review. Of the 22 ongoing studies identified in the review, only four were being conducted in healthcare settings. We have revised the manuscript to reflect this more clearly.
Action Taken: The relevant paragraph in the introduction has been revised to state: "Although the ongoing Cochrane review by Barnes et al. included healthcare settings within its scope, none of the eligible completed studies were conducted in these environments. This may reflect the strict inclusion criteria of the review, which excluded grey literature and non-randomised sources such as government reports, community health programme evaluations, or smoking cessation data not formally published in peer-reviewed journals. Of the 22 ongoing studies identified, only four were being conducted in healthcare settings, with most interventions delivered in schools or via text messaging. This suggests that healthcare environments remain underexplored in the current evidence base for vaping cessation interventions among children and young people."

Reviewer Comment 4: All countries will be considered? Please mention
Response: We will clarify that studies from all countries are eligible.
Action Taken: Added to the methods section: "Studies conducted in healthcare settings from any country will be included to ensure a comprehensive understanding of global practices and interventions."

Reviewer Comment 5: Appendix 1 – Please check spelling of adolescent
Response: "Adolescen*" was intentionally used as a wildcard to capture variations ("adolescent," "adolescence"). We could not identify any other spelling errors.

Reviewer Comment 6: Analysis: Apart from descriptive, subgroup analysis or sensitivity analysis could also be considered
Response: We appreciate this suggestion. We agree that subgroup analysis could offer valuable insights. Therefore, if sufficient data is identified, we will perform subgroup analyses to explore how findings vary across specific groups (e.g., age, healthcare setting, or intervention type).

Action Taken: We have added the following statement to the "Data analysis and presentation" section of the manuscript: "If sufficient data is available, subgroup analyses will be conducted to explore potential variations in findings according to participant characteristics, intervention types, or healthcare settings."

Reviewer Comment 7: Is the scoping review registered?
Response: Thank you for this suggestion. We agree that registration enhances transparency. The protocol will be registered on the Open Science Framework (OSF).

We appreciate the opportunity to revise our manuscript based on your valuable feedback.
